# CAS: enhancing implicit constrained data augmentation with semantic enrichment for biomedical relation extraction and beyond

**DOI:** 10.1093/database/baaf025

**Published:** 2025-07-03

**Authors:** Fang-Yi Su, Gia-Han Ngo, Ben Phan, Jung-Hsien Chiang

**Affiliations:** Department of Computer Science and Information Engineering, National Cheng Kung University, Tainan City 701401, Taiwan; Department of Computer Science and Information Engineering, National Cheng Kung University, Tainan City 701401, Taiwan; Department of Computer Science and Information Engineering, National Cheng Kung University, Tainan City 701401, Taiwan; Department of Computer Science and Information Engineering, National Cheng Kung University, Tainan City 701401, Taiwan

## Abstract

Biomedical relation extraction often involves datasets with implicit constraints, where structural, syntactic, or semantic rules must be strictly preserved to maintain data integrity. Traditional data augmentation techniques struggle in these scenarios, as they risk violating domain-specific constraints. To address these challenges, we propose CAS (Constrained Augmentation and Semantic-Quality), a novel framework designed for constrained datasets. CAS employs large language models to generate diverse data variations while adhering to predefined rules, and it integrates the SemQ Filter. This self-evaluation mechanism ensures the quality and consistency of augmented data by filtering out noisy or semantically incongruent samples. Although CAS is primarily designed for biomedical relation extraction, its versatile design extends its applicability to tasks with implicit constraints, such as code completion, mathematical reasoning, and information retrieval. Through extensive experiments across multiple domains, CAS demonstrates its ability to enhance model performance by maintaining structural fidelity and semantic accuracy in augmented data. These results highlight the potential of CAS not only in advancing biomedical NLP research but also in addressing data augmentation challenges in diverse constrained-task settings within natural language processing.

**Database URL**: https://github.com/ngogiahan149/CAS

## Introduction

**Figure 1.**
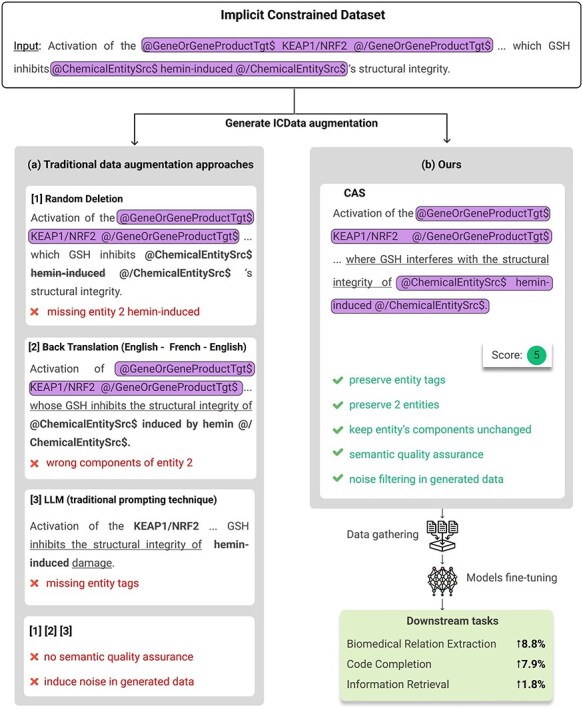
(a) Results of generated data using traditional data augmentation approaches. (b) Results of generated data using our method. Text enclosed in rounded rectangles represents entity tags or components that should be preserved (corresponding to the highlighted text). Text that should have been preserved but was either missing, malformed, or appeared in unexpected positions or with incorrect content is bolded. Underlined text indicates alterations made to the original sentence. Examples on the left demonstrate issues such as missing entities, incorrect tagging, or semantic distortion, while the right shows structurally and semantically consistent results generated by our method.

Data augmentation has proven to be a vital technique for training robust AI models, especially in data-scarce or high-complexity tasks such as biomedical text mining and code completion. By enriching the training set with additional samples or variations, data augmentation can improve model generalization and mitigate overfitting concerns. However, traditional approaches, for example, random deletion [1] or back translation [2], often struggle when applied to highly specialized domains where precise syntactic and semantic constraints must be preserved. Figure 1a illustrates how these methods can inadvertently alter or remove biomedical entities and relationship tags, leading to degraded model performance.

**Figure 2.**
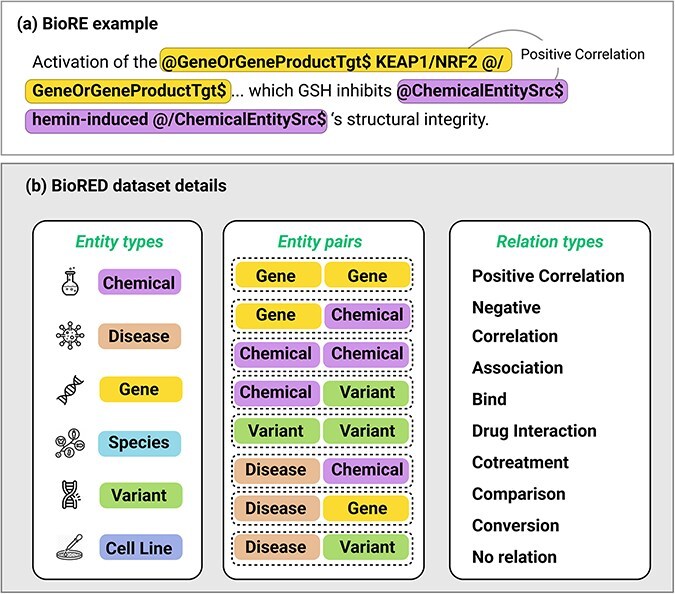
(a) An example of a biomedical relation extraction task for a “Positive Correlation” relation between two types of entity, Gene and Chemical, being wrapped in entity type tagging. There will be a source entity (with “Src” tags) and a Target entity (with “Tgt” tag). The specific tags **@/EntityType$** and **@/EntityType$** should not be changed during the data augmentation. (b) Details of multiple entity types, pairs of entities that may have relation, and multiple relation types.

Many specialized tasks exhibit hidden or implicit constraints on the data structure. For instance, in biomedical relation extraction (BioRE), text must maintain correct entity tags such as @GeneOrGeneProduct and @ChemicalEntity to ensure the integrity of the underlying biomedical knowledge [3]. The detailed structure of these tags, including their syntax and application in BioRE, is fully presented in Fig. 2, which provides an example of a tagged biomedical text alongside an overview of entity types, relation pairs, and relation types based on the BioRED [3] dataset. Similarly, code completion tasks have strict syntax requirements, as altering keywords or functions can cause compilation errors or shift the program’s functionality [1, 4, 5]. Even in information retrieval (IR), question–answer pairs must remain faithful to specific formats, and mathematical reasoning tasks require carefully structured queries to capture multistep logic. A naive data augmentation algorithm may not account for these domain-specific rules, introducing noise and reducing data utility.

To underscore this challenge, we propose the term Implicit Constrained Data (ICData) in which structural, syntactic, or semantic rules cannot be violated without compromising validity. Although no standard term exists for such datasets, our definition highlights the shared difficulty they pose for generic augmentation methods.

In this paper, we introduce CAS (Constrained Augmentation and Semantic-Quality), a two-step framework. First, a Constrained Augmentor generates data variations while preserving domain rules; second, a SemQ Filter discards low-quality or semantically incongruent samples. Demonstrated in biomedical relation extraction (BioRE), code completion, and information retrieval, CAS preserves structure-critical elements and removes problematic augmentations, boosting performance in domains where data integrity is crucial. Figure 1b compares our augmented samples with those from traditional methods.

## Related work

### Data augmentation and its challenges

Previous studies have explored various data augmentation methods to improve generalization in NLP. Classic techniques include random deletion [1], synonym replacement, or back translation [2]. Although these can diversify the training set, they often fail to maintain complex domain-specific requirements. In domains like biomedical text processing, naive approaches may inadvertently disrupt carefully structured entity tags, while in code generation, they risk altering fundamental syntax elements [4, 5].

Recent advancements in data augmentation leverage pretrained language models (PLMs) [6], such as auto-encoders (e.g. BERT), auto-regressive models (e.g. GPT-2), and sequence-to-sequence models (e.g. BART) with label conditioning. Previous work [6] demonstrates that prepending class labels to text sequences is an adequate label conditioning method for PLMs. While these approaches are promising, especially for low-resource domains, they face challenges with constrained datasets. For instance, generating biomedical text that adheres to strict formatting and factual accuracy is difficult. A key challenge lies in balancing the diversity of augmented data with its consistency with the original data’s distribution. Overemphasis on either can hurt downstream performance. Ensuring the augmented data reflects the original distribution without introducing biases is also critical in sensitive domains like healthcare. While BERT excels at label preservation, GPT-2 requires more context for better label consistency. BART, trained with a denoising objective, performs well in low-resource settings. However, methods like text-infilling can replicate biases. The ABEX method [7] addresses some of these issues using Abstract Meaning Representation (AMR) graphs to generate and mix abstract descriptions for diversity. However, ABEX’s reliance on pretrained AMR-to-Text models and the incomplete AMR parsing process creates challenges in ensuring consistent, realistic variations for constrained data.

### Large language model-based generation and filtering

Recent advances in large language models (LLMs) have led to sophisticated strategies for generating synthetic data. Researchers have examined LLM-based generation for information retrieval, producing synthetic query–document pairs or relevance judgments to enhance IR (Information Retrieval) systems. In parallel, several works have investigated using LLMs to block or filter undesirable outputs [8]. These methods demonstrate the potential of large-scale text generation and curation but often focus on removing offensive or irrelevant text rather than enforcing strict structural rules.

### Domain-specific augmentation for ICData

The challenge intensifies when augmenting datasets with implicit constraints, such as preserving hierarchical tags in biomedical relation extraction or ensuring syntactic correctness in code tasks. Existing domain-oriented approaches, like distant labeling [9, 10] or weak supervision [11, 12], provide large-scale pseudo-labeled data but can introduce significant noise or incorrect annotations when the constraints are not explicitly enforced. Moreover, specific biomedical tasks require carefully annotated entity boundaries (Fig. 2), whereas code tasks demand uncompromised syntax. Our approach builds on these prior works by combining LLM-based generation with a semantic filtering mechanism directly addressing domain-specific formatting and labeling rules.

While related work has shown the viability of data augmentation in various domains, few have tackled the generative and filtering processes in an integrated manner tailored to implicit constrained datasets. Thus, we propose that CAS can close this gap. By emphasizing structural fidelity and semantic accuracy, our method aims to mitigate the pitfalls of existing approaches that fail to preserve domain-critical components systematically.

## Method

**Figure 3.**
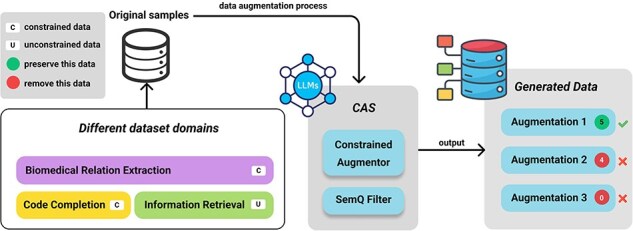
Overall flow of the framework.

Figure 3 presents an overview of our framework. We divide the process into two main steps: data generation using the Constrained Augmentor and subsequent quality assurance with the SemQ Filter. Our primary motivation is to tackle ICData by enforcing domain-specific rules (e.g. preserving entity tags in biomedical text or maintaining valid syntax in code) while introducing meaningful variability.

### Constrained Augmentor

The Constrained Augmentor is a structured framework designed to generate realistic variations of data samples while strictly adhering to domain-specific constraints. This augmented utilizes task-specific prompts to guide generative LLMs in creating data that preserves essential structural, syntactic, and semantic rules, such as entity tags in biomedical texts or syntax tokens in programming tasks. Specifically, the Constrained Augmentor enforces structural constraints by embedding explicit rules into the prompting process, ensuring that generated samples inherently respect domain-specific formats like maintaining balanced entity tags in biomedical texts or correct syntax in code. Importantly, this component does not perform any filtering; its sole purpose is to produce augmentations that comply with predefined structural requirements during the generation phase. Given an original dataset containing $n$ samples ${D_o} = \left\{ {{d_1},{d_2}, \ldots ,{d_n}} \right\}$, we create an augmented dataset ${D_a}$ by applying a prompt-based generative model under explicit format requirements:


(1) $${D_a} = \,\left\{ {Constrained\,Augmentor\left( {{d_i}} \right)\,|\,{d_i}\, \in {D_o}\,} \right\}$$

**Figure 4.**
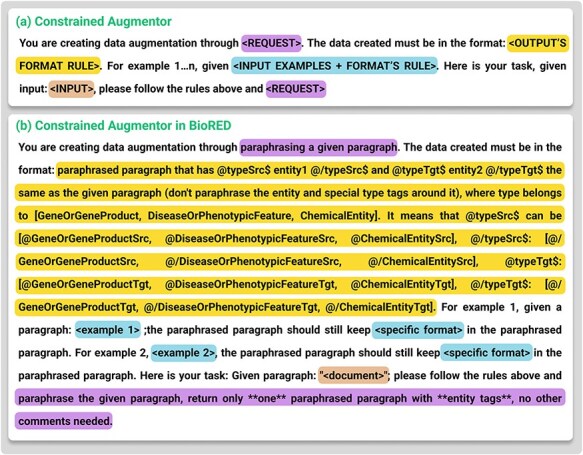
(a) The template for Constrained Augmentor. (b) Details of how Constrained Augmentor is applied in the BioRED dataset.

Where ***Constrained Augmentor*** is stated in Fig. 4a, this augmentor is implemented as a specialized prompt instructing the LLM to modify each sample in permissible ways, such as paraphrasing text while preserving essential tags and structures. For biomedical data (e.g. BioRED), it ensures each pair of @GeneOrGeneProduct or @ChemicalEntity tags is retained. For code tasks, it forbids altering reserved keywords or syntax-critical tokens. By explicitly defining these rules, the Constrained Augmentor differs from generic prompting techniques (e.g. zero-shot) by incorporating task-specific, structured prompts that explicitly encode domain rules, ensuring consistency and quality in constrained data augmentation.

We performed preliminary manual checks on randomly sampled outputs to gauge the reliability of our prompt-based approach. We iteratively refined the prompt instructions whenever recurring pattern errors (e.g. missing closing tags or conflicting code tokens) were observed.

### SemQ Filter

After generating ${D_a}$, we perform a self-evaluation step called the SemQ Filter. The “SemQ Filter” (Semantic Quality Filter) is a self-evaluation mechanism that assigns quality scores to augmented data samples based on domain-specific semantic criteria. Its primary goal is to ensure that generated data samples maintain fidelity to the original dataset’s meaning and intent while enhancing quality and consistency. Notably, the SemQ Filter relies on the language model’s understanding of the text to evaluate semantic fidelity, assessing how well-augmented samples preserve the original content’s meaning without requiring manual enumeration of all possible rules. This distinguishes it from traditional rule-based filtering and makes it adaptable across domains, while the Constrained Augmentor ensures structural integrity during generation. Formally, for each $d_i^{\mathrm{^{\prime}}} \in {D_a}$, the SemQ Filter computes,



_(2)_
$$\begin{aligned}Score\left( {d_i^{\prime}} \right) = SemQ\,Filter\left( {d_i^{\prime}} \right)\;{\mathrm{for each}}\;d_i^{\prime} \in {D_a}\end{aligned}$$

where the SemQ Filter is also prompt-based. It checks for balanced entity tags, correct code syntax, or other domain-specific constraints and assesses whether the paraphrased content preserves the original meaning. Documents scoring below a chosen threshold are excluded, giving us the following:


(3) $$\begin{aligned}{D_{filtered\,}} = \,\left\{ {d_i^{\prime}\, \in \,{D_a}\,|\,Score\left( {d_i^{\prime} > 4} \right)} \right\}\end{aligned}$$

For biomedical relation extraction (BioRE), the Constrained Augmentor ensures the preservation of critical entity tags (e.g. @GeneOrGeneProduct and @ChemicalEntity), while the SemQ Filter evaluates the semantic correctness of the augmented samples. Using task-specific LLM prompts, the SemQ Filter assigns quality scores to augmented samples, discarding those below a predefined threshold (score < 4).

To justify the selection of a threshold score of 5, we analyzed the results shown in Table 1, which evaluates the factuality of LLM-generated responses based on datasets augmented with varying SemQ Filter thresholds. In this context, factuality scores are derived from RefChecker [13], which classifies LLM outputs into three categories:

**Entailment**: Responses directly supported by or consistent with the reference data.

**Contradiction**: Responses that conflict with or refute the reference data.

**Neutral**: Responses that cannot be verified or contradicted due to insufficient information in the reference.

**Table 1.** The analysis of factuality metrics (entailment, contradiction, and neutral) for LLM-generated responses was conducted using RefChecker with the Mixtral 8x7B baseline model. The evaluation involved 1,000 samples, with exactly 200 randomly selected samples for each SemQ Filter score (ranging from Score 1 to Score 5). The results indicate that setting the SemQ Filter threshold to Score 5 during data augmentation significantly enhances semantic fidelity and factual consistency, as evidenced by higher entailment rates and a reduction in contradiction and neutrality.

Threshold score	Entailment (%)	Contradiction (%)	Neutral (%)
Score 1	48.88	0.27	50.86
Score 2	63.48	0.09	36.43
Score 3	73.30	0.75	25.95
Score 4	74.67	1.18	24.14
**Score 5**	**83.10**	**0.78**	**16.12**

The table demonstrates that semantic fidelity and factual consistency improve as the threshold increases. Specifically, Score 5 achieves the highest entailment rate (83.10%) while significantly reducing neutral classifications (16.12%) and contradiction classifications (0.78%). These findings indicate that Score 5 effectively filters out low-quality augmentations while retaining semantically coherent samples.

To validate these results, we tested 1000 samples (200 for each score) using RefChecker [13] with the Mixtral 8x7B [14] baseline model. The analysis confirms that Score 5 offers the best balance between preserving semantic alignment and minimizing noise, making it an optimal choice for downstream tasks requiring high factual accuracy. This threshold ensures that augmented datasets contribute to generating LLM responses with enhanced factual consistency and semantic fidelity.

**Figure 5.**
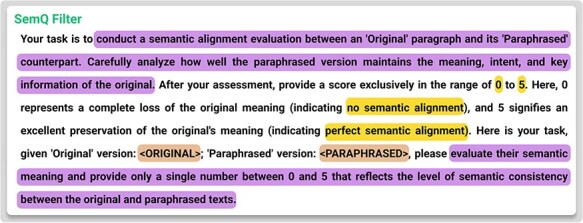
Template for SemQ filter.

Figure 5 provides an example of the prompting template used for this filtering process. Consequently, (4) emerged as a balanced cutoff. The final augmented dataset ${D_{{\mathrm{final}}}}$ is then:


(4) $${D_{final\,}} = \,{D_o}\,\mathop \cup \,{D_{filtered}}$$

Combining the Constrained Augmentor’s targeted generation with the SemQ Filter’s selective scrutiny, we effectively address the twin objectives of injecting diversity while preserving domain-critical formats. Figures 4b and 5 detail the specific prompt templates for biomedical relation extraction tasks, illustrating how the system enforces tagging rules and evaluates consistency.

## Experimental settings

We evaluate CAS on multiple tasks encompassing biomedical relation extraction, code completion, mathematical reasoning, and information retrieval. Below, we describe the datasets, model configurations, and additional details relevant to each experiment.

### Dataset

**BioRED dataset** [3]: The task is to extract biomedical relations across multiple entity types with multiple relation types. At the document level, numerous entity types exist (gene, chemical, disease, etc.) and relation pairs (disease–gene, chemical–chemical). There are 600 PubMed abstracts, with 400 documents for training, 100 for dev, and 100 for validation. The BioRED dataset generates data augmentations, fine-tuning models, and evaluating model performance.

**EvolInstruct-80k-v1 dataset** (https://huggingface.co/datasets/nickrosh/Evol-Instruct-Code-80k-v1): EvolInstruct-80k-v1 dataset (license cc-by-nc-sa-4.0) is an open-source dataset generated using Evol-Teacher [15]. The dataset contains about 78 000 instructions and their code solution. We use this dataset to create data augmentation and fine-tune the model for the Code Generation task.

**HumanEval dataset** [16]: HumanEval contains 164 handwritten Python programming problems with a function signature, docstring, body, and several unit tests. It focuses on testing the execution ability of the code using the evaluation metric pass@k. This dataset is used as a testing set for the Code Generation task.

**MultiPL-E dataset** [17]: MultiPL-E is a benchmark for evaluating large language models for code generation that supports 18 programming languages. It takes the OpenAI “HumanEval” Python benchmark and uses little compilers to translate them into other languages. This dataset is also used as a testing set for the Code Generation task. In this paper, we do experiments on C++, Java, JavaScript (JS), R, Bash, and Rust.

**Grade School Math 8 K (GSM8K) dataset** [18]: GSM8k is a dataset of 8.5 K high-quality linguistically diverse grade school math word problems. The dataset was created to support the task of answering questions on fundamental mathematical problems that require multistep reasoning.

**Natural Questions (NQ) dataset** [19]: The NQ corpus contains real user questions issued to Google search and answers found from Wikipedia by annotators. NQ is designed to train and evaluate automatic question-answering systems. This dataset is used for creating data augmentation, training, and testing.

### Experimental design

**Data augmentation**: We use open-source LLM OpenChat-3.5 [20] as the Constrained Augmentor and SemQ Filter model. As stated in Wang *et al*. [20], OpenChat-3.5 performs comparable to ChatGPT-3.5 in multiple benchmarks.

**Biomedical relation extraction**: The BioRED dataset is used for training and evaluation. We fine-tune PubMedBERT [21] over 50 epochs with a learning rate of 1e-5 and a batch size of 10. To evaluate the effectiveness of CAS, we compare it with several state-of-the-art baselines, including PubMedBERT and BERT-GT [3] were selected as they are recent works specifically applied to the BioRED dataset, demonstrating their relevance and strong performance in biomedical relation extraction. ATLOP [22] and ATLOP (AFL) (i.e. an enhanced version of ATLOP that replaces the original adaptive thresholding loss with an adaptive focal loss [23]) were chosen due to their demonstrated effectiveness on BioRED dataset in recent studies. Additionally, PLM-CE, PLM-SupCon, PLM-MCCL, MTB-CE, MTB-SupCon, and MTB-MCCL from Zhou *et al*. [24] were included as they represent state-of-the-art augmentation approaches applied to biomedical datasets, providing a robust benchmark for evaluating CAS. Since the paper only reported F1-scores, Precision and Recall are not included in our experiments.

Additionally, we consider recent data augmentation techniques to contextualize the advantages of CAS further. The ABEX method [7], for instance, employs an “Abstract-and-Expand” paradigm that utilizes AMR graphs to generate abstract descriptions and subsequently expands them into synthetic data using a fine-tuned BART model [6]. This approach is innovative in balancing diversity and consistency in augmentations but lacks mechanisms to ensure factuality.

Transformer-based data augmentation methods [6], as outlined in studies utilizing models like BERT, GPT-2, and BART, offer another perspective by focusing on conditional data generation techniques such as masking tokens and prepending class labels to input sequences. These methods excel in low-resource settings but target syntactic variation and class label preservation. In contrast, CAS emphasizes semantic fidelity by integrating a self-evaluation mechanism to filter out low-quality augmentations, addressing the critical need for domain-specific accuracy and quality in biomedical datasets.

By situating CAS within the broader landscape of data augmentation strategies, we highlight its ability to address the limitations of existing methods while aligning with the specific demands of biomedical relation extraction tasks.

**Code generation**: For fine-tuning our model, we utilized the EvolInstruct-80k dataset, selecting Deepseek-Coder-1.3b-Instruct [25] as the foundational model. The model is fine-tuned in 800 steps, with a learning rate of 2e-5. We employed datasets including HumanEval, MultiPL-E, and GSM8K for evaluation purposes. Since there is a difference between the performances stated in Guo *et al*. [25] and those obtained through our use of the bigcode-evaluate-harness [26], we opted to rely on the performance evaluations derived from bigcode-evaluate-harness for all models to ensure consistency in comparative analysis. Temperature is set to 0.2 for all models, and the primary metric used for evaluating model performance in this task was pass@1. Our model’s performance was benchmarked against several others, including CodeGeeX2 6B [27], StarCoder 15B [28], CodeLlama 7B [29], CodeLlama-Instruct 7B [29], and CodeLlama 13B [29].

**Mathematics reasoning**: We additionally assess the influence on the performance of a model that has been fine-tuned for the code generation task when applied to a different task. This involves comparing a model already fine-tuned for code generation with various other LLMs, including Llama 7B [30], Llama 13B [30], Minerva 8B [31], CodeGeeX2 7B [27], StarCoder 15B [28], and Deepseek-Coder-1.3b-Instruct [25].

**Figure 6.**
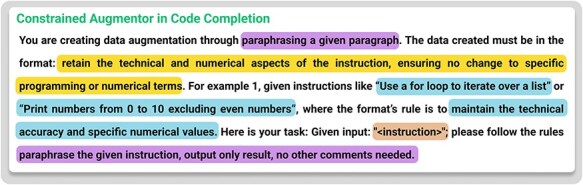
Constrained Augmentor applied in information retrieval task.

**Information retrieval**: For the IR task, the Constrained Augmentor is designed to preserve specific terms critical for relevance, such as query-specific keywords or contextually important terms, while allowing for rephrasing or expansion of surrounding content. The prompt templates explicitly encode these constraints (Fig. 6), ensuring that term integrity is maintained. We fine-tune three models, GPT-2 [31], Llama-2 7B [32] and GPT-J [33], utilizing LoRA fine-tuning. The hyperparameters are rank *r* = 8, alpha = 8, 3 epochs, and learning rate = 5e-5. Each model undergoes fine-tuning with and without integrating data augmentation to assess the impact of CAS on the original model.

**Analysis of label noise and entity tag preservation**: To further investigate how CAS handles the dual challenges of label integrity and structural correctness, we design a small-scale manual evaluation on a subset of augmented samples. First, we randomly select instances from the BioRED dataset to check for label noise, specifically monitoring whether newly generated relationships remain consistent with the original annotation guidelines (e.g. correct gene–chemical pairings). Second, we examine entity tag preservation, ensuring crucial markers like @GeneOrGeneProduct or @ChemicalEntity remain intact after augmentation. We log and quantify any deviations from the expected formatting or semantics, allowing us to assess how the Constrained Augmentor and SemQ Filter collectively mitigate errors. The findings of this analysis are presented in Section “Analysis of label noise and entity tag preservation”.

## Results and discussion

### Generated data and success rate

Table 2 provides a comprehensive comparison of the number of successfully generated samples across three challenging domains: biomedical relation extraction (BioRE), code generation (Evol Instruct), and Information Retrieval (NQ) before and after applying the SemQ Filter. This filter evaluates the semantic quality of generated data to ensure consistency and accuracy. The success rate, defined as the proportion of generated data that passes through the SemQ Filter, is calculated as:


(5) $$\begin{aligned}& Success\,\,Rate\,\,of\,\,Generated\,\,Data\,\,Passing\,\,SemQ\,\,Filter\!\nonumber \\ &\quad =\! \,\frac{{Sem\! - \!Ori}}{{Aug\! -\! Ori}}\end{aligned}$$

where “Aug-Ori” represents the total number of generated augmentations, and “Sem-Ori” denotes the number of augmentations retained after semantic evaluation.

**Table 2.** Data augmentation in different dataset domains

Dataset	Ori	Aug	Sem	Success rate of generated data passing SemQ Filter (%)
**BioRED**	28 233	87 519	60 188	53.9
		(+59 286)	(+31 955)	
**Evol Instruct**	78 381	156 762	133 568	70.4
		(+78 381)	(+55 187)	
**NQ**	396 000	496 715 (+100 715)	418 826 (+43 818)	43.5

Ori: Original, Aug: After Augmentation, Sem: After semantic filtering. The line separates constrained datasets (above), generated by detailed examples to the model, and unconstrained datasets (below), generated without given examples to the model.

**Figure 7.**
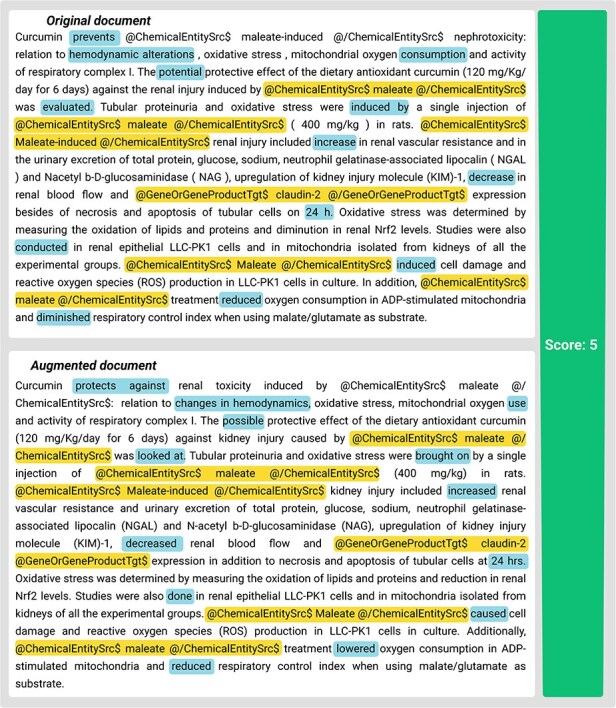
Example of generated data in BioRED with semantic score. Highlights indicate modifications that preserve the same semantic meaning. Underlines denote the entities with unique tags around them.

**Figure 8.**
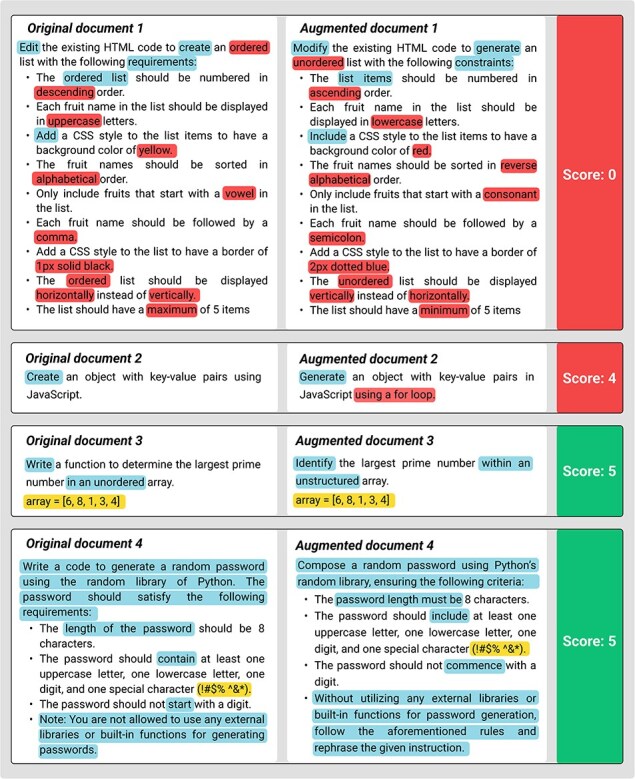
Example of generated data in Code Completion with semantic score. Highlights indicate modifications that preserve the same semantic meaning. Underlines denote the entities with unique tags around them. Bold text represents modifications that alter the semantic meaning.

The results reveal notable differences across domains. In the biomedical relation extraction task, the data augmentation process involves intricate manipulation of text containing rigorously formatted tags, such as @GeneOrGeneProduct and @ChemicalEntity, requiring precise placement and pairing of these markers. As shown in Figs 7 and 8, the SemQ Filter rigorously scrutinizes the congruence of these tags, resulting in a relatively lower success rate due to the stringent criteria necessary to maintain scientific accuracy. Despite these challenges, the filtered dataset exhibits improved consistency, verified through small-scale manual checks.

**Figure 9.**
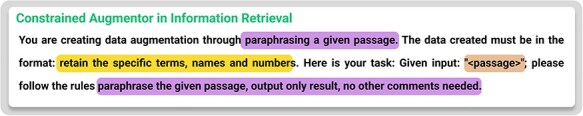
Constrained Augmentor applied in code completion task.

In contrast, the code generation task shows a higher success rate post-augmentation. The augmentation process in this domain ensures that syntactic structures and numerical values remain intact, preserving the original logic and functionality of the code (Fig. 9). This adaptability to the programming context enables the generation of syntactically valid and semantically coherent code snippets, aligning with the original intent of the code author. The results underscore the effectiveness of our method in handling the nuances of programming languages.

The success rate for the Information Retrieval task dropped to ∼44%. Unlike the biomedical and programming tasks, this decline can be attributed to the absence of structured examples with clear rules for augmentation (Fig. 6). This lack of predefined guidelines significantly hampers the augmentation process, emphasizing the importance of providing clear and consistent example rules to achieve optimal performance.

These findings highlight the challenges faced in different domains, underscoring the need for context-aware augmentation strategies to navigate the nuanced linguistic, syntactic, and semantic rules inherent in specialized datasets.

### BioRED experimental results

**Comparison with baselines**: Table 3 illustrates the performance of our approach, “CAS,” under three evaluation schemas: entity-pair extraction, relation-type identification, and novelty characterization. CAS refers to PubMedBERT trained with augmented data ${D_o}\mathop \cup {D_{{\mathrm{filtered}}}}$. Notably, CAS consistently outperforms standard PubMedBERT and other baselines. Specifically, CAS demonstrates a marked improvement, including an 8.8% enhancement in the F1-score, highlighting its robustness in handling the complexities of biomedical data. The results underscore CAS’s ability to excel in extracting entity pairs within a relation, discerning relation types, and characterizing the novelty of entity pairs [3].

**Table 3.** Evaluation results on Subtask 2 at BioCreative VIII when augmented data are added: extracting the entity pairs within a relation, second schema: extracting the entity pairs and the relation type, and third schema: further labeling the novelty for the extracted pairs

Eval. schema	Methods	All	<G, D>	<G, G>	<G, C>	<D, V>	<C, D>	<C, V>	<C, C>
	BERT-GT	72.1	63.8	78.5	77.7	69.8	76.2	58.5	74.9
**Entity pair**	PubMedBERT	72.9	67.2	78.1	78.3	**67.9**	76.5	**58.1**	78.0
	CAS	**78.3**	**76.1**	**86.2**	**85.7**	66.0	**78.2**	38.1	**85.9**
		↑ 5.4	↑ 8.9	↑ 7.1	↑ 7.4	↓ 1.9	↑ 1.7	↓ 20	↑ 7.9
	BERT-GT	56.5	54.8	63.5	60.2	42.5	67.0	11.8	52.9
**+Relation type**	PubMedBERT	58.9	56.6	66.4	59.9	**50.8**	65.8	**25.8**	54.4
	CAS	**64.4**	**64.3**	**76.1**	**64.3**	47.0	**72.2**	9.5	**59.7**
		↑ 5.5	↑ 7.7	↑ 9.7	↑ 4.4	↓ 3.8	↑ 6.4	↓ 16.3	↑ 5.3
	BERT-GT	44.5	37.5	47.3	55.0	36.9	51.9	11.8	48.5
**+Novelty**	PubMedBERT	47.7	40.6	54.7	54.8	42.8	51.6	**12.9**	50.3
	CAS	**56.5**	**51.1**	**69.1**	**57.9**	**44.9**	**78.2**	9.5	**56.5**
		↑ 8.8	↑ 10.5	↑ 14.4	↑ 3.1	↑ 2.1	↑ 26.6	↓ 3.4	↑ 6.2

All numbers are *F*-scores. The <G, D> is the concept pair of the gene (G) and the disease (D). The columns of those entity pairs present the RE performance in F1-scores. Bold denotes the best model.

Table 4 comprehensively compares various models’ performance on the BioRED test set, evaluating their Precision, Recall, and F1 scores. Among all the models listed, CAS emerges as the best-performing model, achieving the highest scores: Precision (70.1), Recall (59.6), and F1-score (64.4). CAS significantly outperforms many state-of-the-art models, including PLM-CE, MTB-SupCon, ATLOP, TriA-BioRE, etc. CAS effectively handles constrained biomedical data, often involving intricate relationships and domain-specific challenges. These results affirm the effectiveness of this approach in extracting meaningful relationships within biomedical datasets.

**Table 4.** Evaluation of different models on the BioRED test set

Model	Precision	Recall	F1-scores
**PLM-MCCL**	-	-	55.1
**PLM-SupCon**	-	-	52.2
**PLM-CE**	-	-	57.7
**MTB-CE**	-	-	61.5
**MTB-SupCon**	-	-	43.1
**MTB-MCCL**	-	-	60.8
**ATLOP**	49.5	45.8	47.6
**ATLOP (AFL)**	55.2	44.1	49.1
**TriA-BioRE**	61.8	42.4	50.3
**CAS**	**70.1**	**59.6**	**64.4**

Performance metrics include Precision, Recall, and F1-scores. Bold denotes the best models

**Minimal pair comparisons**: To further evaluate the generalizability of CAS, we tested its augmentation on PubMedBERT results provided in Table 5, observing similar gains in Recall and F1-scores. This demonstrates that CAS is architecture-agnostic and can adapt effectively to different baseline models. These findings validate the versatility of CAS and its potential to enhance a broad range of relationship extraction frameworks.

**Table 5.** Comparison of data augmentation methods for subtask 2 at BioCreative VIII using fine-tuned PubMedBERT

Data augmentation method	Entity pair				Entity pair +relation type				Entity pair +novelty		
	P	R	F		P	R	F		P	R	F
ABEX	74.35	72.49	73.41		54.21	52.85	53.52		57.65	56.20	56.91
Pretrained transformer	73.32	**77.25**	75.23		52.87	55.70	54.25		55.78	**58.68**	**57.24**
CAS (our method)	**85.24**	72.48	**78.35**		**70.07**	**59.59**	**64.41**		**61.48**	52.28	56.51

Table 5 presents a comparative analysis of three data augmentation methods for the BioRE task: ABEX [7], Pretrained Transformer [6], and CAS. CAS serves as the unifying framework in this setup, integrating each augmentation method with its Constrained Augmentor (CA) for structured generation and SemQ Filter for semantic quality control. The table reports Precision, Recall, and F1 scores across different evaluation criteria, demonstrating how each augmentation method performs when enhanced by the CAS framework. The results show that combining our baseline augmentation approach with CAS achieves the highest performance, underscoring the framework’s ability to improve augmentation outcomes. Specifically, in entity pair extraction, CAS surpasses ABEX by ∼5% and the Pretrained Transformer by around 3% in F1-score. When considering entity pairs and relation type identification, CAS substantially improved, outperforming ABEX by ∼11% and the Pretrained Transformer by around 10%.

However, CAS performs slightly worse for entity pair and novelty characterization, with F1-scores lower than ABEX by ∼0.4% and the Pretrained Transformer by around 0.7%. The somewhat lower performance of CAS in the entity pair +novelty criterion can be attributed to its focus on constrained data generation and semantic filtering, prioritizing precision and consistency over novelty. This strict filtering may inadvertently remove data with novel relationships, better captured by methods like ABEX’s “Abstract-and-Expand” paradigm or the Pretrained Transformer. The Pretrained Transformer, fine-tuned on the BioRED dataset, leverages its pretraining and fine-tuning capabilities to explore diverse relationships and capture novelty more effectively, albeit at the cost of precision in some scenarios. In contrast, CAS excels in tasks requiring precision and relational understanding, as its two-step approach effectively filters out noisy or semantically inconsistent data. This makes CAS particularly suitable for applications where data quality and accuracy are paramount.

### Code generation and mathematical reasoning

As demonstrated in Table 6, “Deepseek-Coder-Instruct + CAS” significantly enhances code completion accuracy across multiple programming languages, with notable improvements of 5.0% in Java and 7.0% in C++, highlighting the effectiveness of CAS in leveraging the underlying LLM for superior data augmentation. These results illustrate CAS’s capability to produce substantial gains in code generation tasks.

**Table 6.** Evaluation results on completion task The evaluation metric is pass@1. Bold denotes the best model. A demarcation line is placed between the final two models to emphasize our primary objective: to compare the cement our method brings relative to the base mode

Model	Size	HumanEval	MultiPL-E					
			C++	Java	R	Rust	Bash	JS
**CodeGeeX2**	6B	33.5	29.2	23.5	6.8	17.9	6.3	24.8
**StarCoder**	16B	30.4	28.6	28.5	13.6	21.2	9.3	31.7
**CodeLlama**	7B	30.0	25.5	29.2	16.8	26.3	8.1	31.8
**CodeLlama-Instruct**	7B	45.7	8.7	28.8	14.9	26.3	8.7	33.1
**CodeLlama**	13B	35.1	34.1	32.2	**22.4**	**28.2**	**11.8**	38.3
**Deepseek-Coder Instruct**	1.3B	51.8	29.2	31.0	12.4	23.6	7.5	39.1
**CAS**	1.3B	**53.7**	**34.2**	**38.0**	13.7	24.4	11.2	**39.1**
		**↑ 1.9**	**↑ 5.0**	**↑ 7.9**	**↑ 1.3**	**↑ 0.8**	**↑ 3.7**	**↑ 0.0**

l. The table evaluates the performance of three approaches: ABEX, Pretrained Transformer, and CAS (our method) across three evaluation criteria: Entity Pair, Entity Pair + Relation Type, and Entity Pair + Novelty. Precision (P), Recall (R), and F1-score (F) are reported for each criterion.

For mathematics reasoning on GSM8K, Table 7 shows that CAS, despite being fine-tuned exclusively on a code instruction dataset without explicit mathematical content, retains commendable resilience with only a marginal decrease in accuracy. While fine-tuning on code tasks does not directly optimize for solving multistep word problems, the results suggest minimal negative transfer to unrelated tasks. This slight dip in performance underscores the importance of task-aligned data for fine-tuning and thoroughly exploiting the model’s capabilities in domain-specific tasks. Nonetheless, the findings highlight the model’s flexibility and provide valuable insights into the transferability of learned representations across diverse contexts.

**Table 7.** Performance on math reasoning dataset

Model	Size	Accuracy
**Llama**	7B	11.0
**Llama**	13B	17.8
**Minerva**	8B	16.2
**CodeGeeX2**	7B	22.2
**StarCoder**	16B	23.4
**Deepseek-Coder-Instruct**	1.3B	**34.9**
**CAS**	1.3B	30.0

Bold denotes best performance.

### Information retrieval

Table 8 presents an empirical analysis comparing the outcomes of training models with and without data augmentation, highlighting moderate yet consistent improvements across Llama-2, GPT-2, and GPT-J when the CAS (Contextualized Augmentation Strategy) is applied to IR data. While the exact match (EM) score gains vary, the general trend underscores that carefully filtering out semantically incoherent samples and augmenting them with high-quality data can benefit IR tasks. Notably, the degree of improvement differs among models, with those exhibiting higher baseline performance, such as Llama-2, achieving the most significant enhancements among the three models.

**Table 8.** Performance on information retrieval task

Model	Size	EM (%)
**Llama-2**	7B	32.38
**Llama-2 + CAS**	7B	34.18
		↑ 1.8
**GPT-2**	1.5B	9.58
**GPT-2 + CAS**	1.5B	9.61
		↑ 0.03
**GPT-J**	6B	31.44
**GPT-J + CAS**	6B	31.99
		↑ 0.55

Bold denotes best performance.

### Analysis of label noise and entity tag preservation

We examined label noise (incorrect relational labels, misclassified entity types) and entity tag preservation (damage or removal of @GeneOrGeneProduct, @ChemicalEntity, etc.). Random checks on 100 augmented BioRED samples revealed around 8% label misalignment, which often stemmed from semantic inconsistencies that the SemQ Filter identified and removed during its evaluation of semantic fidelity. However, the Constrained Augmentor minimizes such issues by enforcing structural rules during generation, reducing the initial occurrence of misaligned labels. Meanwhile, roughly 11% initially displayed broken tags. Still, these structural errors were largely mitigated by the Constrained Augmentor’s design, with <2% of the final retained samples exhibiting tag-related issues after the SemQ Filter’s semantic assessment. These analyses confirm the complementary roles of the Constrained Augmentor and the SemQ Filter in preserving domain-critical components.

## Limitations

The effectiveness of CAS is closely tied to the performance of the underlying large language models used in both augmentation and filtering. Additionally, while we selected a threshold of 4 based on empirical testing, different domains or tasks may benefit from a data-driven or adaptive threshold approach. Furthermore, the SemQ Filter, while effective at removing low-quality augmentations, may inadvertently discard some high-quality instances that do not fully meet its semantic criteria. Future work could address this limitation by refining the filter’s scoring or adding validation steps to minimize the loss of valuable data.

## Conclusions and future work

We introduce a novel approach to ICData augmentation, complemented by a self-evaluative mechanism. By defining “ICData” as datasets with strict structural or semantic rules ranging from entity tagging in biomedical texts to syntactic constraints in code, we highlight the difficulties that naive augmentation methods encounter in such domains. CAS, comprising the Constrained Augmentor and the SemQ Filter, consistently improves performance in biomedical relation extraction, code generation, and IR tasks. Compared to traditional methods, CAS autonomously evaluates and ensures the semantic quality of augmented data, reducing the need for manual intervention. Due to computational resource constraints, our evaluation of augmentation strategies and the SemQ Filter’s impact was limited to selected baselines and experiments. Future work will extend this analysis to include a broader range of LLM-based augmentation methods and systematically study the interplay between the Constrained Augmentor and the SemQ Filter.

## Data Availability

The data underlying this article are available in various repositories and can be accessed via DOI links. The BioRED dataset is available at https://doi.org/10.1093/bib/bbac282. The EvolInstruct-80k-v1 dataset can be found on https://huggingface.co/datasets/nickrosh/Evol-Instruct-Code-80k-v1 The HumanEval dataset is accessible through https://doi.org/10.48550/arXiv.2107.03374, while the MultiPL-E dataset is hosted by https://doi.org/10.48550/arXiv.2110.14168. Additionally, the Grade School Math 8K (GSM8K) dataset is available on https://doi.org/10.48550/arXiv.2110.14168, and the Natural Questions (NQ) dataset can be accessed via https://doi.org/10.1162/tacl_a_00276.
